# The effects of N-acetylcysteine supplement on metabolic parameters in women with polycystic ovary syndrome: a systematic review and meta-analysis

**DOI:** 10.3389/fnut.2023.1209614

**Published:** 2023-09-29

**Authors:** Jiajun Liu, Haodong Su, Xueshan Jin, Lan Wang, Jieming Huang

**Affiliations:** ^1^The First Affiliated Hospital of Guangzhou University of Chinese Medicine, Guangzhou, China; ^2^The First Clinical Medical College of Guangzhou University of Chinese Medicine, Guangzhou, China; ^3^College of Traditional Chinese Medicine, Jinan University, Guangzhou, China

**Keywords:** polycystic ovary syndrome, N-acetylcysteine, metabolic parameters, nutritional supplementation, meta-analysis

## Abstract

**Objectives:**

Polycystic ovary syndrome (PCOS) is a common endocrine disease, often accompanied by metabolic disorders. Metformin, as an insulin sensitizer, is widely used to improve the metabolic function of PCOS, but may have gastrointestinal side effects. Emerging evidence suggests that N-acetylcysteine (NAC) improves metabolic parameters in PCOS and may be a potential alternative to metformin.

**Methods:**

We searched four online databases, PubMed, Embase, Web of Science, and Cochrane Library, from inception to April 1, 2023. The *I*^2^ statistic and Cochrane’s Q test were employed to determine heterogeneity between studies, with an *I*^2^ value >50% or *p* < 0.1 considered significant. The data were expressed as standardized mean differences and corresponding 95% confidence intervals.

**Results:**

A total of 11 randomized controlled trials were included in the final analysis, including 869 women with PCOS. The results showed that NAC caused more changes in body mass index (SMD: −0.16, 95% CI: −0.40 to 0.08), body weight (SMD: −0.25, 95% CI: −0.50 to 0.00), fasting insulin (SMD: −0.24, 95% CI: −0.53 to 0.06), ratio of fasting blood glucose to fasting insulin (SMD: 0.38, 95% CI: −0.33 to 1.08), total cholesterol (SMD: −0.11, 95% CI: −0.39 to 0.17), triglycerides (SMD: −0.18, 95% CI: −0.63 to 0.28), and low-density lipoprotein (SMD: −0.09, 95% CI: −0.51 to 0.33) compared with metformin. Compared with metformin or placebo, NAC significantly reduced fasting blood-glucose levels (SMD: −0.23, 95% CI: −0.43 to −0.04; SMD: −0.54, 95% CI: −1.03 to −0.05, respectively). In addition, NAC significantly reduced total cholesterol (SMD: −0.74, 95% CI: −1.37 to −0.12), and this effect was observed when NAC was compared with placebo. However, NAC reduced HDL levels in women with PCOS compared with metformin (SMD: −0.14, 95% CI: −0.42 to 0.14).

**Conclusion:**

This study suggests that NAC is effective in improving metabolic parameters in PCOS and may be a promising nutritional supplement for the treatment of PCOS.

**Systematic review registration:**https://www.crd.york.ac.uk/PROSPERO/display_record.php?RecordID=415172, identifier CRD42022339171.

## Introduction

Polycystic ovary syndrome (PCOS) is the most common endocrine disorder in women of reproductive age, affecting approximately 6–13% of women (1). The 2003 Rotterdam consensus is widely used in clinical practice as the diagnostic criteria, mainly including the following three items: anovulation or rare ovulation; elevated androgen; ovarian polycystic changes (2). The clinical manifestations of PCOS are diverse, including menstrual disorders, infertility, obesity, hirsity, acne, and mood disorders (3). It also increases the risk of cardiovascular disease (CVD), type 2 diabetes, and metabolic syndrome, all of which have long-term effects (4). Because PCOS mostly affects young women (premenopausal women), many treatments focus on immediate signs and symptoms rather than the long-term effects of PCOS (5). The prevalence of metabolic syndrome in women with PCOS is high, reaching 33% (6). Another study also reported that adolescent women with PCOS had a 3-fold increased risk of metabolic syndrome compared with healthy adolescent women (7). Metabolic syndrome is related to a high risk of CVD, hypertension, and certain psychological disorders (6). Early intervention of metabolic disorders in PCOS women has important clinical implications.

Insulin resistance is an important feature of PCOS, which is independent of obesity and body fat distribution, and is also frequently reported in lean patients (8–10). In PCOS patients, insulin-sensitive tissues such as liver, skeletal muscle, and fat lose their sensitivity to insulin, while the ovaries remain highly sensitive to insulin (10–12). Insulin directly stimulates the production of androgens by cells lining the ovary. Insulin-like growth factor (IGF-1) acts synergistically with luteinizing hormone, and hyperinsulinemia increases the binding site of LH and the androgen response to LH (10, 13). Insulin resistance independently increases the activity of CYP17A1, an enzyme that produces testosterone and androstenedione (12). The effect of insulin on body adipose tissue is another important pathogenesis of PCOS. Insulin stimulates fat production and inhibits fat decomposition, which leads to the accumulation of fatty acids (12). Insulin resistance causes an increase in plasma free fatty acid levels, which affects the liver and adipose tissue (14).

Nutritional supplement therapy as a natural and low-risk adjunct in the treatment of PCOS has attracted more and more attention from clinicians and patients (15). In a large observational study among U.S. adults, dietary supplements use has been stable over the past 10 years, with 52% of participants admitting to using at least one dietary supplement in the previous month and 10% admitting to using at least four (16). N- acetylcysteine (NAC) is an acetylated variant of the L-cysteine amino acid, scavenging reactive oxygen species, is considered a safe, effective and economical nutritional supplement (17). The application of NAC in the clinical treatment of PCOS was first reported by Fulghesu et al. (18), and there have been increasing studies since then (18). A meta-analysis of 18 studies involving 2,185 women with PCOS that evaluated the potential impact of NAC on sex hormones and ovulation reported that NAC significantly reduced total testosterone levels and may be positive in improving reproductive system function in women with PCOS (17). Similarly, another meta-analysis also showed that NAC significantly improved ovulation and pregnancy rates in women with PCOS. Unfortunately, no meta-analysis has reported the relationship between NAC supplement and metabolic parameters in women with PCOS (19). Currently, the relationship between the two is mainly derived from RCTs, so it is necessary to summarize relevant studies to obtain quantitative data between NAC and PCOS women’s metabolism.

This study aimed to comprehensively review published population-based RCTs to evaluate the effect of NAC on metabolic parameters in women with PCOS and to obtain preliminary quantitative data on this effect by meta-analysis.

## Methods

### Study design

This systematic review and meta-analysis was performed in accordance with PRISMA (Preferred Reporting Items for Systematic Reviews and Meta Analyses) guidelines (20). The study has been registered on PROSPERO under the registration number CRD42023415172.

### Search strategy

Two investigators independently searched four online databases, PubMed, Embase, Web of Science, and Cochrane Library, based on medical subject headings (Mesh) and text terms, from inception to April 1, 2023. The following terms were combined in the search strategy: (1) terms related to PCOS (“Polycystic ovarian syndrome” and “PCOS”); (2) terms related to NAC (“Acetylcysteine” and “N-Acetylcysteine” and “NAC”); (3) terms related to RCTs (“Randomized controlled trial” and “Placebo” and “RCT”). In addition, in order to find all eligible articles, we manually searched the references listed in the published meta-analyses. Detailed search strategy can be obtained in Supplementary Table S1.

### Study selection

The following PICO (Population, Intervention, Comparison and Outcome) elements were set as inclusion criteria: (1) Population: women with PCOS; (2) Intervention: PCOS women with oral NAC; (3) Comparison group: PCOS women with oral placebo; (4) Outcome: Outcomes of interest were metabolic parameters such as body mass index (BMI), fasting blood glucose (FBG), fasting insulin (FI), total cholesterol (TC), triglyceride (TG), low-density lipoprotein (LDL), high-density lipoprotein (HDL). Studies were excluded when they were the following. (1) Reviews, case reports, conference abstracts, and cell experiments. (2) Full text was not available or data could not be extracted. (3) NAC combined with other nutritional supplements or drugs was used as the intervention group. In addition, when the same study was reported in multiple different articles, we selected the one that provided the most detailed data.

### Data extraction and quality assessment

Two investigators independently performed data extraction and quality assessment for the final included literature, and any disagreements would be determined by a third investigator. We included the following data: first author name, year of publication, region of study, the sample size in the trial group and the control group, the ages of PCOS women, the doses of NAC and metformin, the duration of follow-up, and the effect size of the baseline and post-intervention outcome measures.

The Cochrane Risk of Bias tool was used to assess the quality of the included studies. Studies that met four or more of the seven criteria were considered low risk, and the remainder were high risk. Detailed scores for each article are provided in Supplementary Table S2.

### Statistical analyses

We extracted the mean and the corresponding standard deviation (SD) of the changes in the data before and after treatment from the literature, and if data on the changes were not directly available, we extracted the relevant data at baseline and after the intervention, and calculated them by the following formula: $\begin{array}{l} \;\mathrm{\Delta mean}=mean_{\mathrm{end}}-mean_{\mathrm{baseline}}\;\\ \Delta SD=\sqrt{SD_{\mathrm{end}}^{2}+SD_{\mathrm{baseline}}^{2}-\;2\times R\times SD_{\mathrm{end}}\times SD_{\mathrm{baseline}}} \end{array}$ (21). The *I*^2^ statistic and Cochrane’s Q test were employed to determine heterogeneity between studies, with an *I*^2^ value >50% or *p* < 0.1 considered significant. Due to the clinical and methodological heterogeneity among studies, a random effect model was used to combine the data. Because the study was not conducted in the same population and the units of metabolic measures were not uniform, the results are expressed as standardized mean differences (SMD) with 95% confidence intervals (CI). Different confounding factors (e.g., duration of intervention, NAC dose, degree of obesity) may have influenced the results. We performed subgroup analyses of BMI, FBG, and FI according to BMI stratification, duration of the intervention, and study region. Egger’s test was performed to determine publication bias. All analyses were performed with the use of Stata software, version 15.1 (Stata Corporation, College Station, TX, United States).

## Results

### Study selection

**Figure 1:**
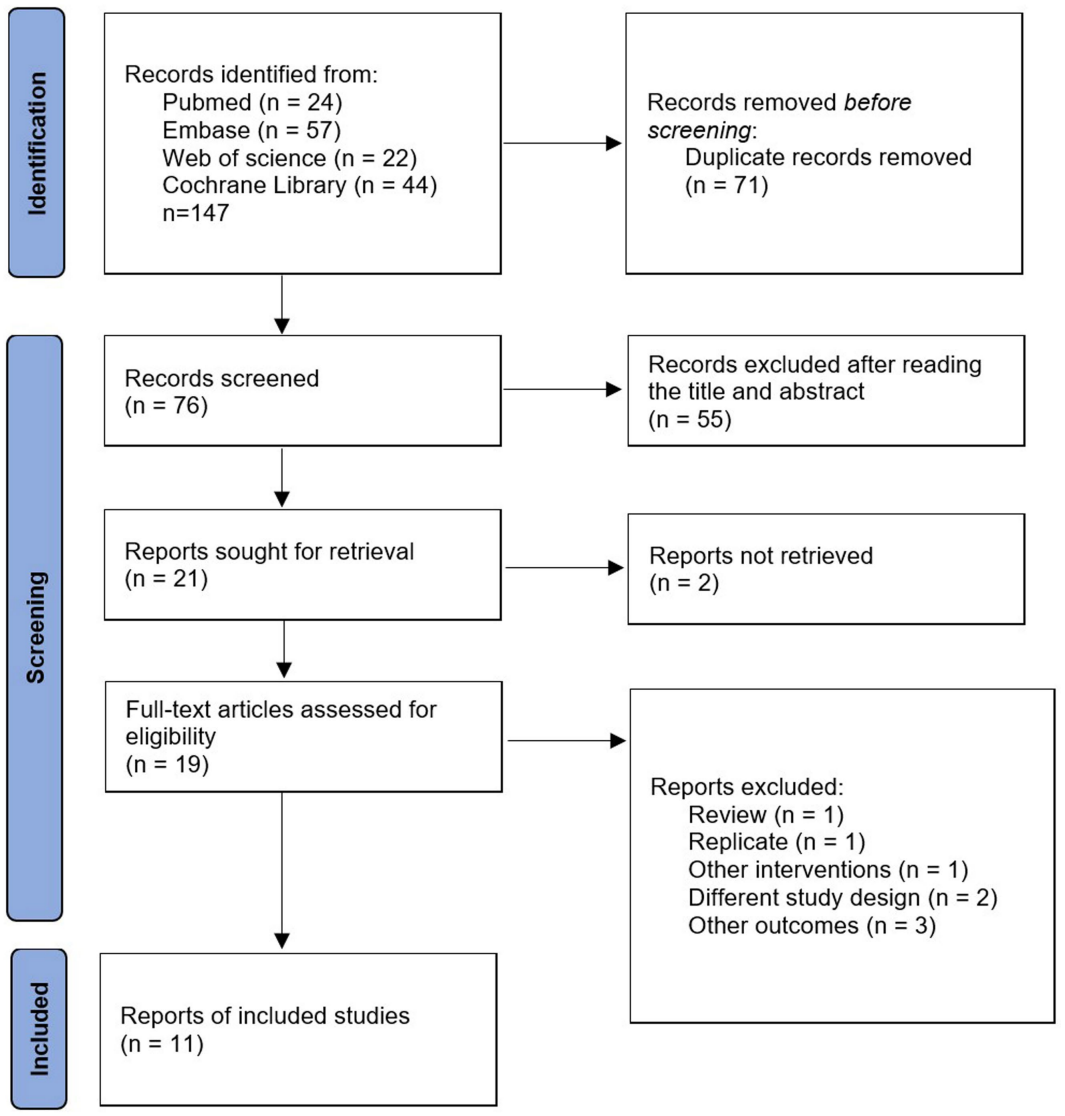
Flowchart for searching and selection details.

We searched four online databases for 147 relevant articles, including 71 duplicates. Of the remaining 76 articles, 55 were excluded after reading the titles and abstracts, leaving a total of 21 articles eligible for full text reading. Ten studies were excluded for the following reasons: full text was not retrieved (*n* = 2); review (*n* = 1); duplicate articles (*n* = 1); other interventions (*n* = 1); different experimental designs (*n* = 2); other outcome indicators (*n* = 3). Finally, 11 studies were included in the study. Detailed information on inclusion and exclusion is available in Figure 1.

### Study characteristics

Overall, we included 11 RCTs involving 869 women with PCOS, published between 2009 and 2022 (22–32). Of these studies, the majority were conducted in Asia and only 1 was conducted in Africa (30), of which 5 were conducted in India (25, 26, 28, 29, 32), 4 in Iran (22, 24, 27, 31), 1 in Egypt (30), and 1 in Turkey (23). Most studies had a control group medication of metformin, one had a placebo (oral rehydration salts) (22), and one was a three arm study with a control group medication of placebo and metformin (24). All study intervention groups received NAC at a dose of 1,500 mg/d and were followed up for 6–24 weeks. The majority of studies diagnosed PCOS according to the Rotterdam consensus, and information on the diagnosis of PCOS was not available for only one study (25). Details of study characteristics can be obtained from Table 1.

**Table 1:** Selected characteristics of eleven studies.

Study	Country	PCOS definition	Sample size		Age (Mean ± SD)		Intervention		Follow-up Duration	Main outcomes
			Trial	Control	Trial	Control	Trial	Control		
Arya 2022	India	Rotterdam*	50	50	27.86 ± 5.66	28.66 ± 5.19	NAC 600 mg, tid	MET 500 mg, tid	24 weeks	Weight, BMI, FBG, FI, FBG/FI
Chandil 2018	India	Rotterdam	45	45	26.82 ± 5.42	27.64 ± 5.11	NAC 600 mg, tid	MET 500 mg, tid	24 weeks	Weight, BMI, FBG, FI, FBG/FI
Cheraghi 2014	Iran	Rotterdam	15	15	29.67 ± 3.35	28.07 ± 3.41	NAC 600 mg, tid	MET 500 mg, tid	6 weeks	Weight, BMI, FBG, FI, TC, TG, LDL, HDL
Elnashar 2007	Egypt	Rotterdam	31	31	27.33 ± 3.35	26.73 ± 5.36	NAC 600 mg, tid	MET 1500 mg/d	6 weeks	BMI, FBG, FI, FBG/FI
Gayatri 2010	India	Rotterdam	50	50	23.2 ± 4.1	22.6 ± 3.8	NAC 600 mg, tid	MET 500 mg, bid/tid	3 months	Weight, BMI, FBG, FI, FBG/FI
Gupta 2017	India	Rotterdam	22	22	Majority of cases (70%) < 30 years		NAC 600 mg, tid	MET 500 mg, bid/tid	3 months	Weight, BMI, FBG, FI, FBG/FI
Javanmanesh 2015	Iran	Rotterdam	46	48	28.98 ± 4.42	29.75 ± 4.90	NAC 600 mg, tid	MET 500 mg, tid	24 weeks	BMI, FBG, FI, TC, HOMA, TG, LDL, HDL
Kumar 2018	India	Not available	50	50	27.1 ± 2.6	26.2 ± 3.2	NAC 600 mg, tid	MET 500 mg, tid	12 weeks	Weight, BMI, FBG, FI, FBG/FI
Nemati 2017	Iran	Rotterdam	54	54	No significant differences**		NAC 600 mg, tid	MET 500 mg, tid	8, 12 weeks	BMI, FBG, FI
Oner 2011	Turkey	Rotterdam	45	30	23.7 ± 4.4	22.6 ± 4.8	NAC 600 mg, tid	MET 500 mg, tid	24 weeks	BMI, FBG, FI, FBG/FI, TC, TG, LDL, HDL
Cheraghi 2014	Iran	Rotterdam	15	15	29.67 ± 3.35	27.93 ± 2.8	NAC 600 mg, tid	ORS, tid	6 weeks	Weight, BMI, FBG, FI, TC, TG, LDL, HDL
Salehpour 2009	Iran	Rotterdam	18	18	27.22 ± 5.35	27.89 ± 6.10	NAC 600 mg, tid	ORS, tid	6 weeks	Weight, BMI, FBG, FI, FBG/FI, TC, TG, LDL, HDL

Rotterdam*: diagnosed by Rotterdam criteria. No significant differences**: patients in both groups have the same variables including age. NAC, N-acetylcysteine; MET, metformin; ORS, oral rehydration salts, used as placebo; tid, three times a day; bid, twice a day; BMI, body mass index; FBG, fasting blood glucose; FI, fasting insulin; FBG/FI, ratio of FBG/FI; TC, total cholesterol; TG, triglyceride; LDL, low-density lipoprotein; HDL, high-density lipoprotein.

### Results for the primary outcome measures

**Figure 2:**
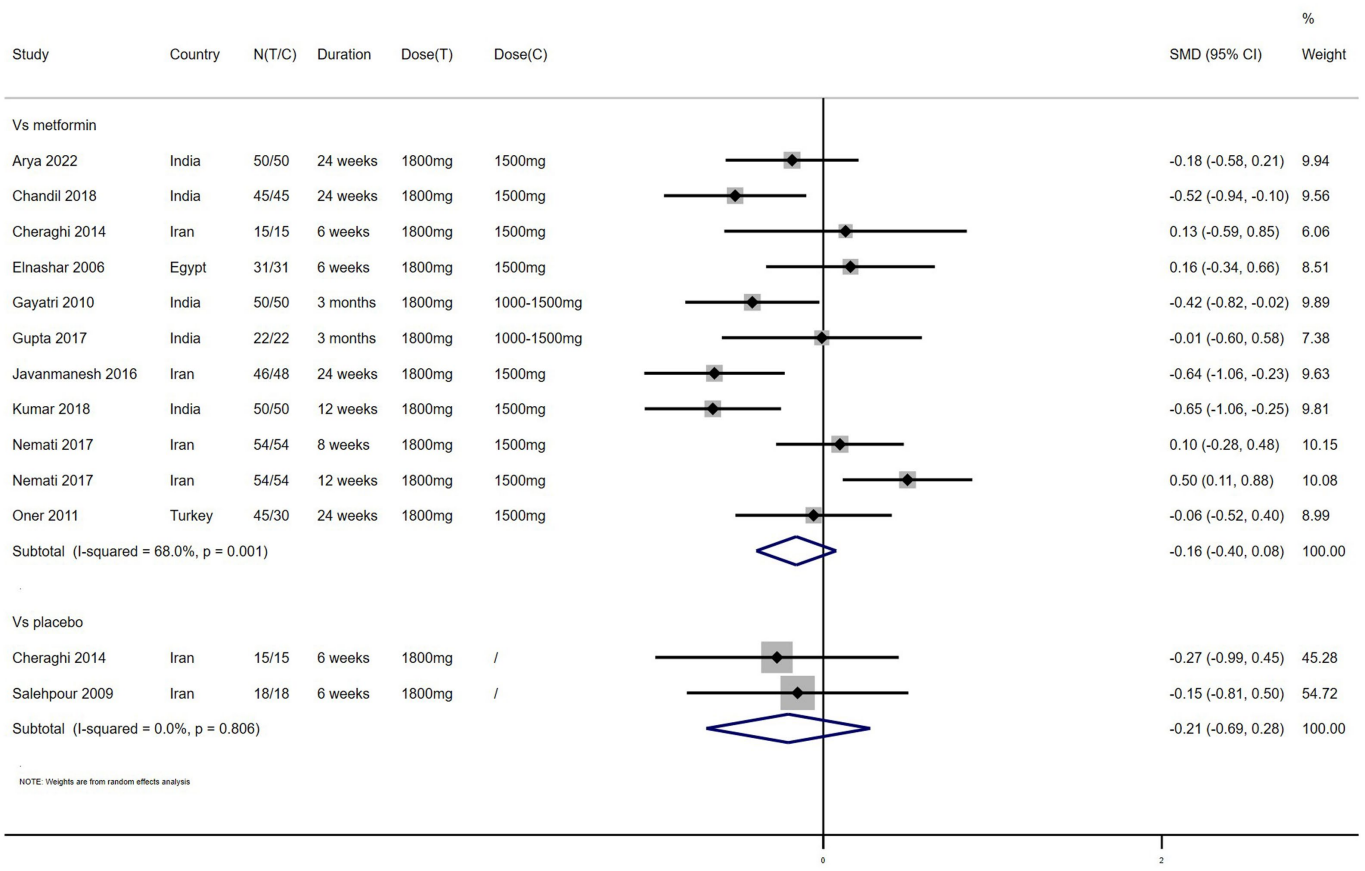
Forest plots of comparing NAC with metformin or placebo on BMI.

BMI – 11 studies reported the effect of NAC on BMI (Figure 2). The results suggested that NAC reduced BMI in women with PCOS, but there was no significant difference when compared with metformin or placebo (SMD: −0.16, 95% CI: −0.40 to 0.08, *I*^2^ = 68.0%, P_H_ = 0.001, *p* = 0.185; SMD: −0.21, 95% CI: −0.69 to 0.28, *I*^2^ = 0, P_H_ = 0.806, *p* = 0.402, respectively).

**Figure 3:**
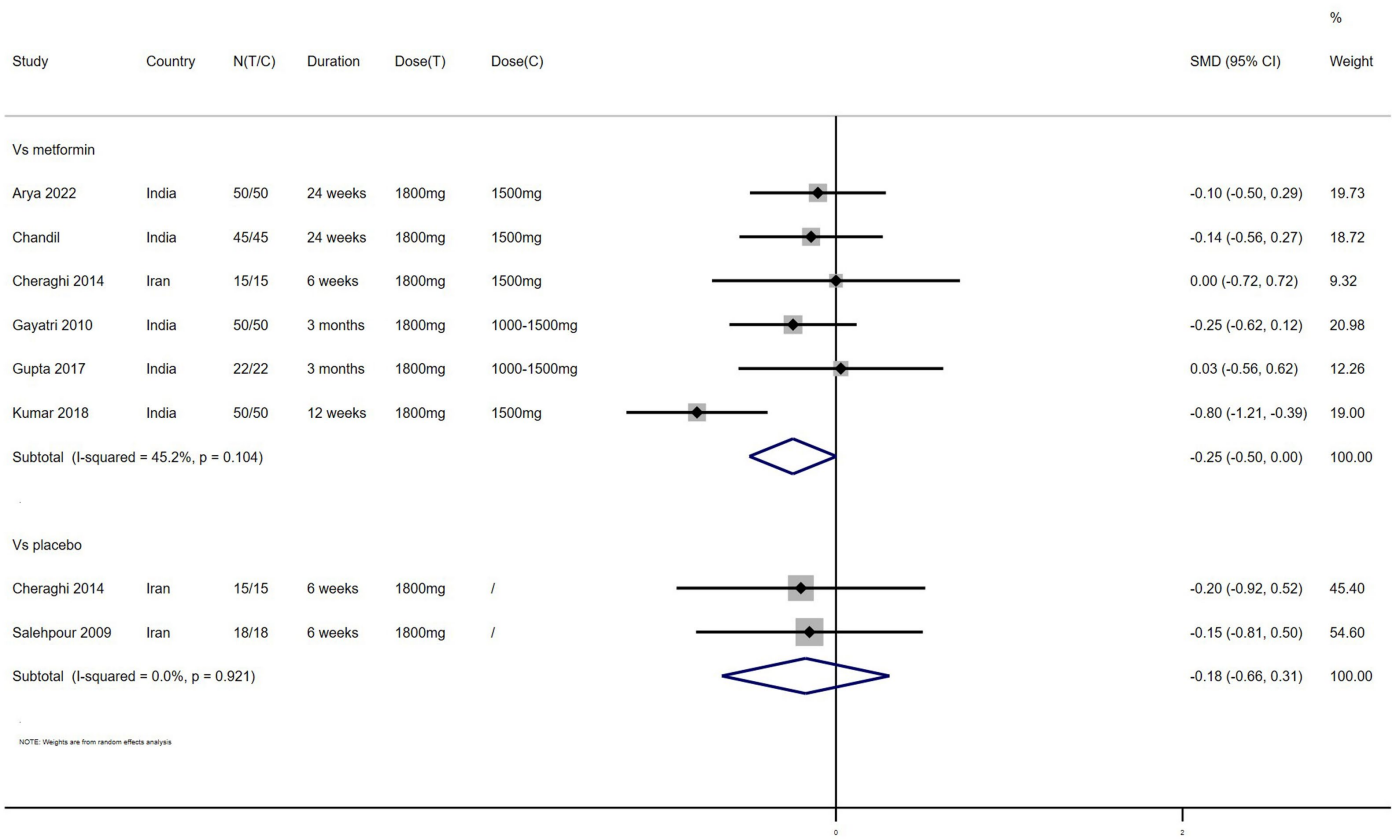
Forest plots of comparing NAC with metformin or placebo on weight.

Weight – 7 studies reported the effect of NAC on weight (Figure 3). The results suggested that NAC reduced weight in women with PCOS, but there was no significant difference when compared with metformin or placebo (SMD: −0.25, 95% CI: −0.50 to 0.00, *I*^2^ = 45.2%, P_H_ = 0.104, *p* = 0.053; SMD: −0.18, 95% CI: −0.66 to 0.31, *I*^2^ = 0, P_H_ = 0.921, *p* = 0.477, respectively).

**Figure 4:**
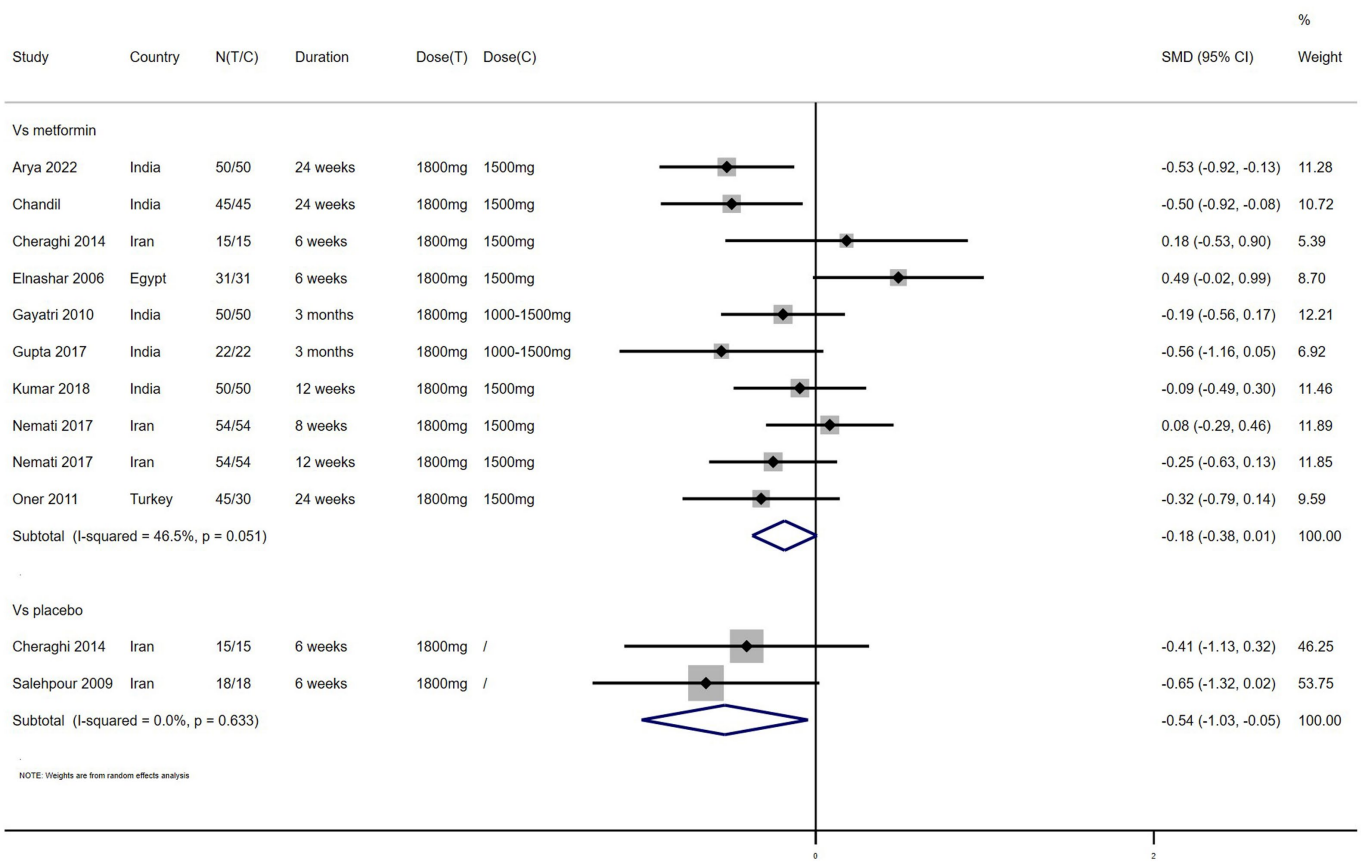
Forest plots of comparing NAC with metformin or placebo on fasting blood glucose (FBG).

FBG – 11 studies reported the effect of NAC on FBG (Figure 4). The results suggested that NAC reduced FBG levels in women with PCOS, and there was a significant difference when compared with metformin or placebo (SMD: −0.23, 95% CI: −0.43 to −0.04, *I*^2^ = 53.5%, P_H_ = 0.018, *p* = 0.02; SMD: −0.54, 95% CI: −1.03 to −0.05, *I*^2^ = 0, P_H_ = 0.633, *p* = 0.032, respectively).

**Figure 5:**
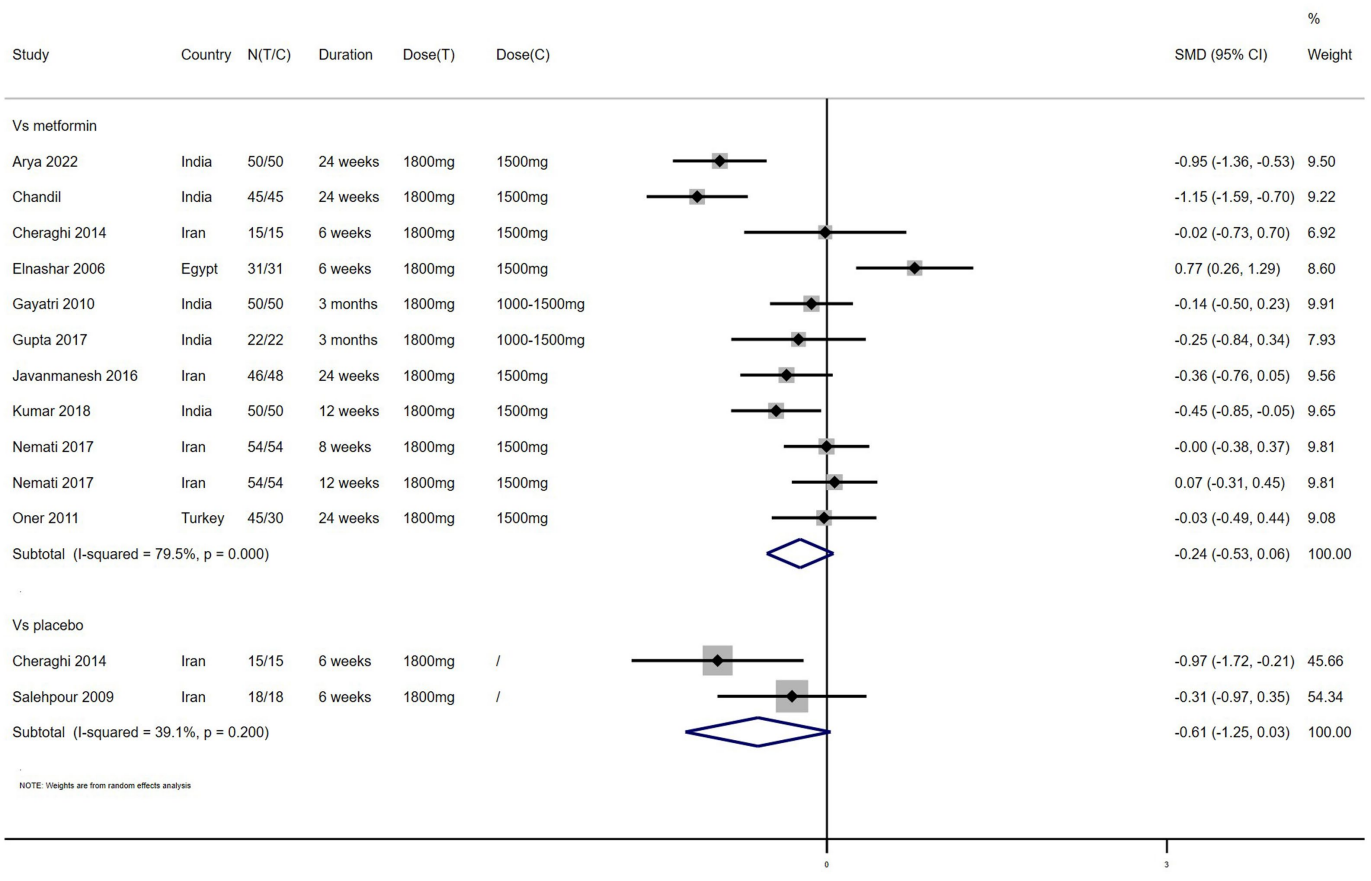
Forest plots of comparing NAC with metformin or placebo on fasting insulin (FI).

FI – 11 studies reported the effect of NAC on FI (Figure 5). The results suggested that NAC reduced FI levels in women with PCOS, but there was no significant difference when compared with metformin or placebo (SMD: −0.24, 95% CI: −0.53 to 0.06, *I*^2^ = 79.5%, P_H_ = 0, *p* = 0.115; SMD: −0.61, 95% CI: −1.25 to −0.03, *I*^2^ = 39.1%, P_H_ = 0.200, *p* = 0.063, respectively).

**Figure 6:**
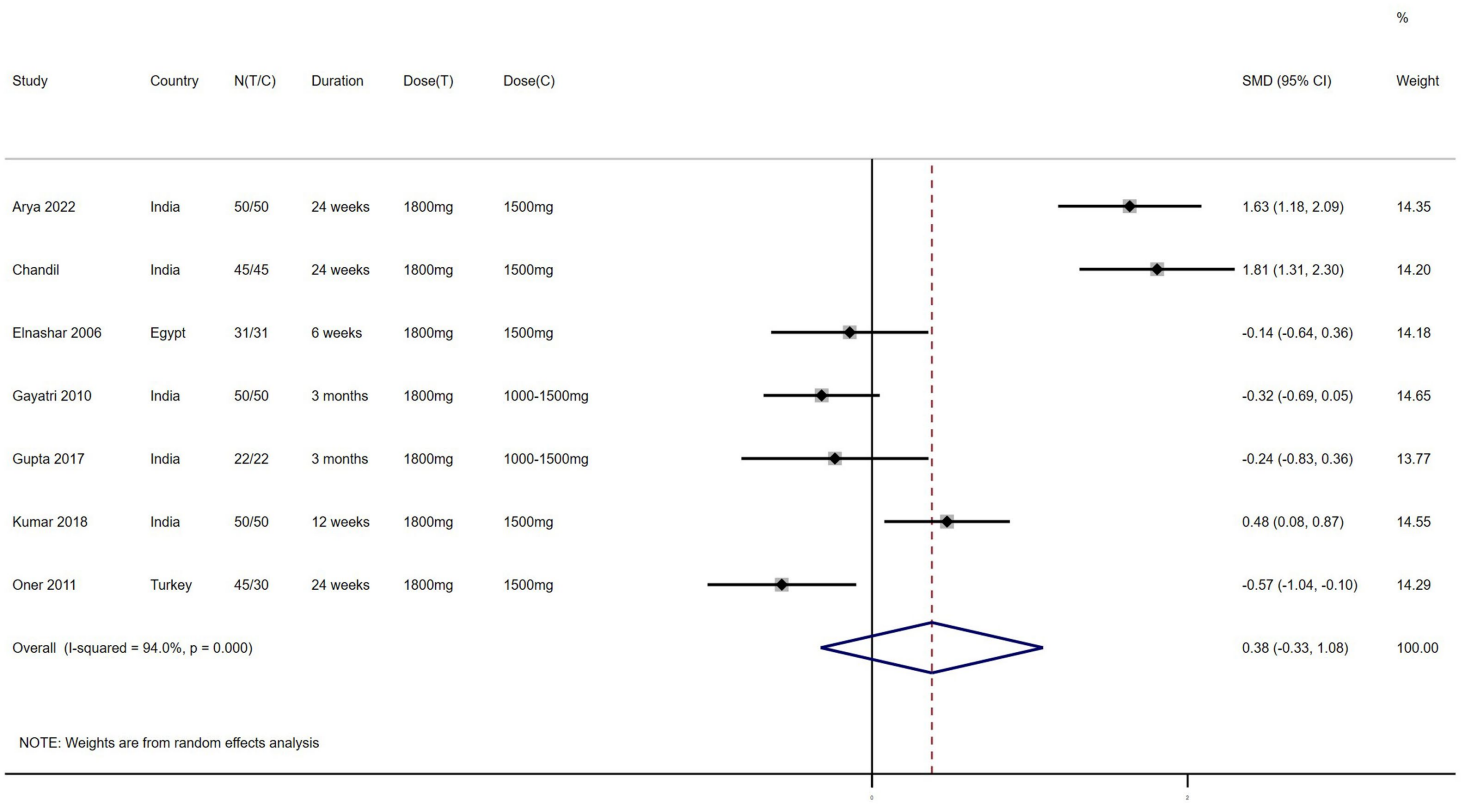
Forest plots of comparing NAC with metformin or placebo on the ratio of fasting blood glucose to fasting insulin (FBG/FI).

FBG/FI – 7 studies reported the effect of NAC on FBG/FI (Figure 6). The results suggested that NAC increased the ratio of FBG/FI in women with PCOS, but there was no significant difference when compared with metformin (SMD: 0.38, 95% CI: −0.33 to 1.08, *I*^2^ = 94.0%, P_H_ = 0, *p* = 0.291).

**Figure 7:**
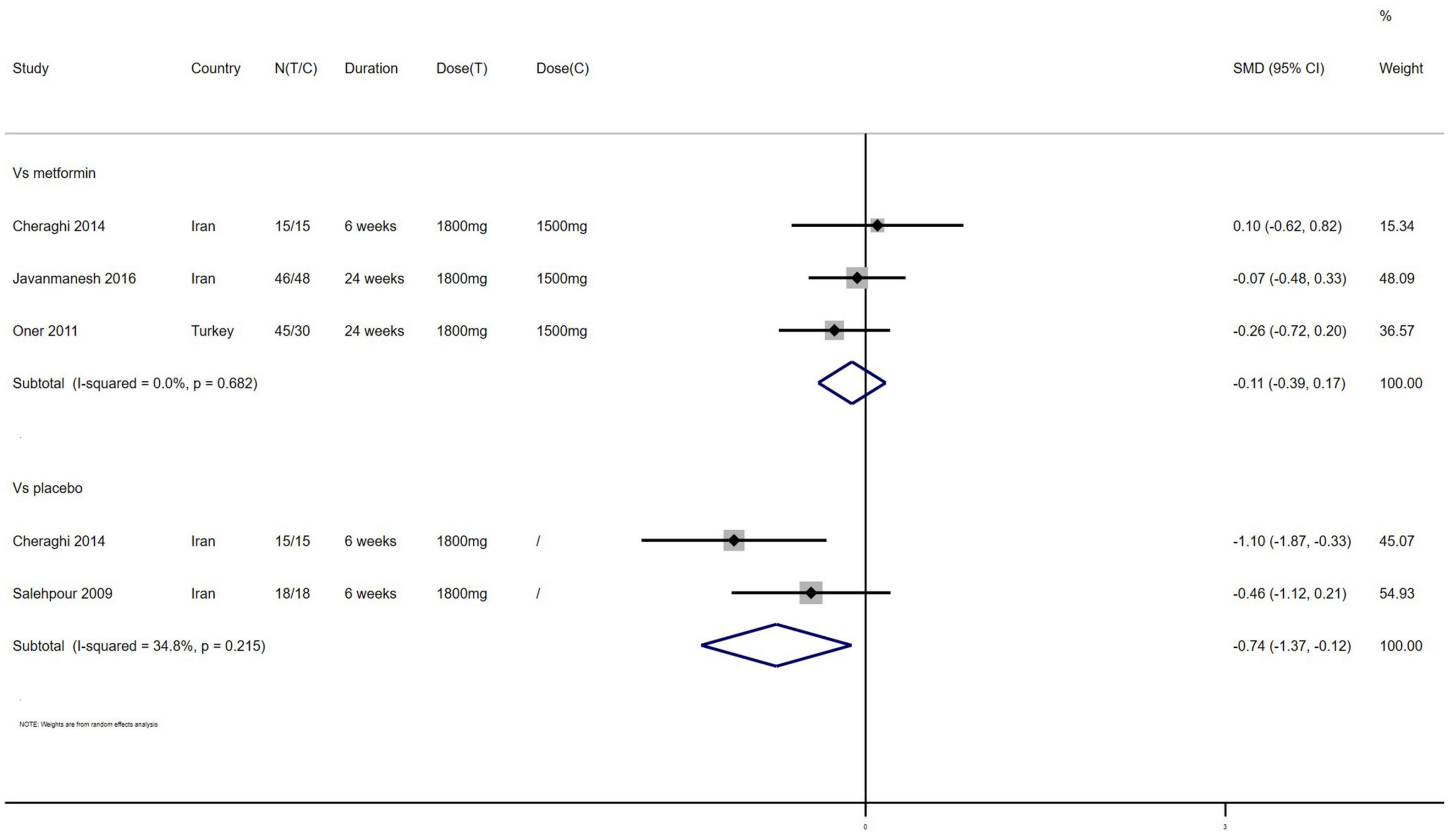
Forest plots of comparing NAC with metformin or placebo on total cholesterol (TC).

TC – 4 studies reported the effect of NAC on TC (Figure 7). The results suggested that NAC reduced TC levels in women with PCOS, and there was significant difference when compared with placebo but not metformin (SMD: −0.74, 95% CI: −1.37 to −0.12, *I*^2^ = 34.8%, P_H_ = 0.215, *p* = 0.020; SMD: −0.11, 95% CI: −0.39 to 0.17, *I*^2^ = 0, P_H_ = 0.682, *p* = 0.426, respectively).

**Figure 8:**
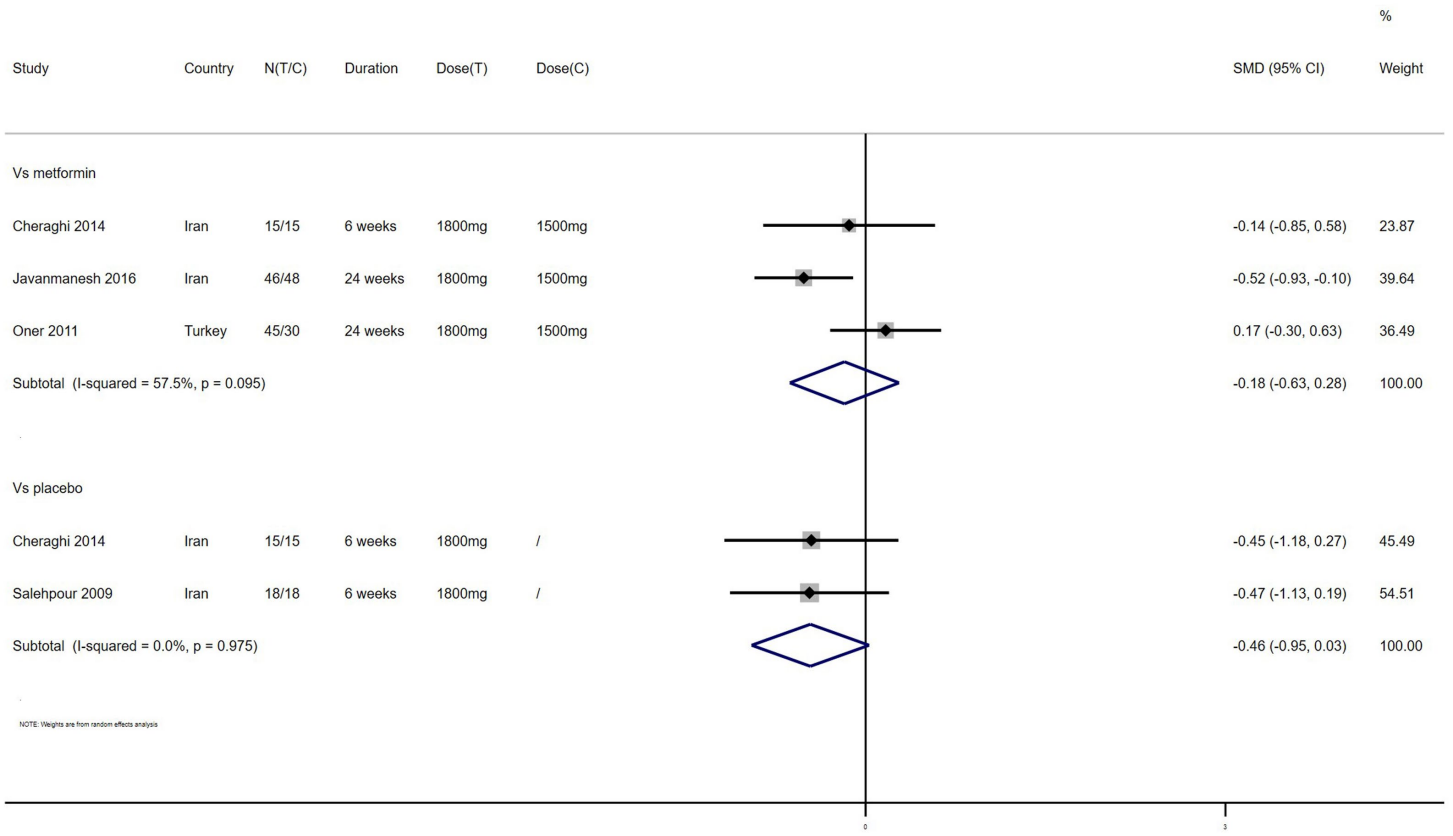
Forest plots of comparing NAC with metformin or placebo on triglyceride (TG).

TG – 4 studies reported the effect of NAC on TG (Figure 8). The results suggested that NAC reduced the level of TG in women with PCOS, but there was no significant difference when compared with metformin or placebo (SMD: −0.18, 95% CI: −0.63 to 0.28, *I*^2^ = 57.5%, P_H_ = 0.095, *p* = 0.446; SMD: −0.46, 95% CI: −0.95 to 0.03, *I*^2^ = 0, P_H_ = 0.975, *p* = 0.065, respectively).

**Figure 9:**
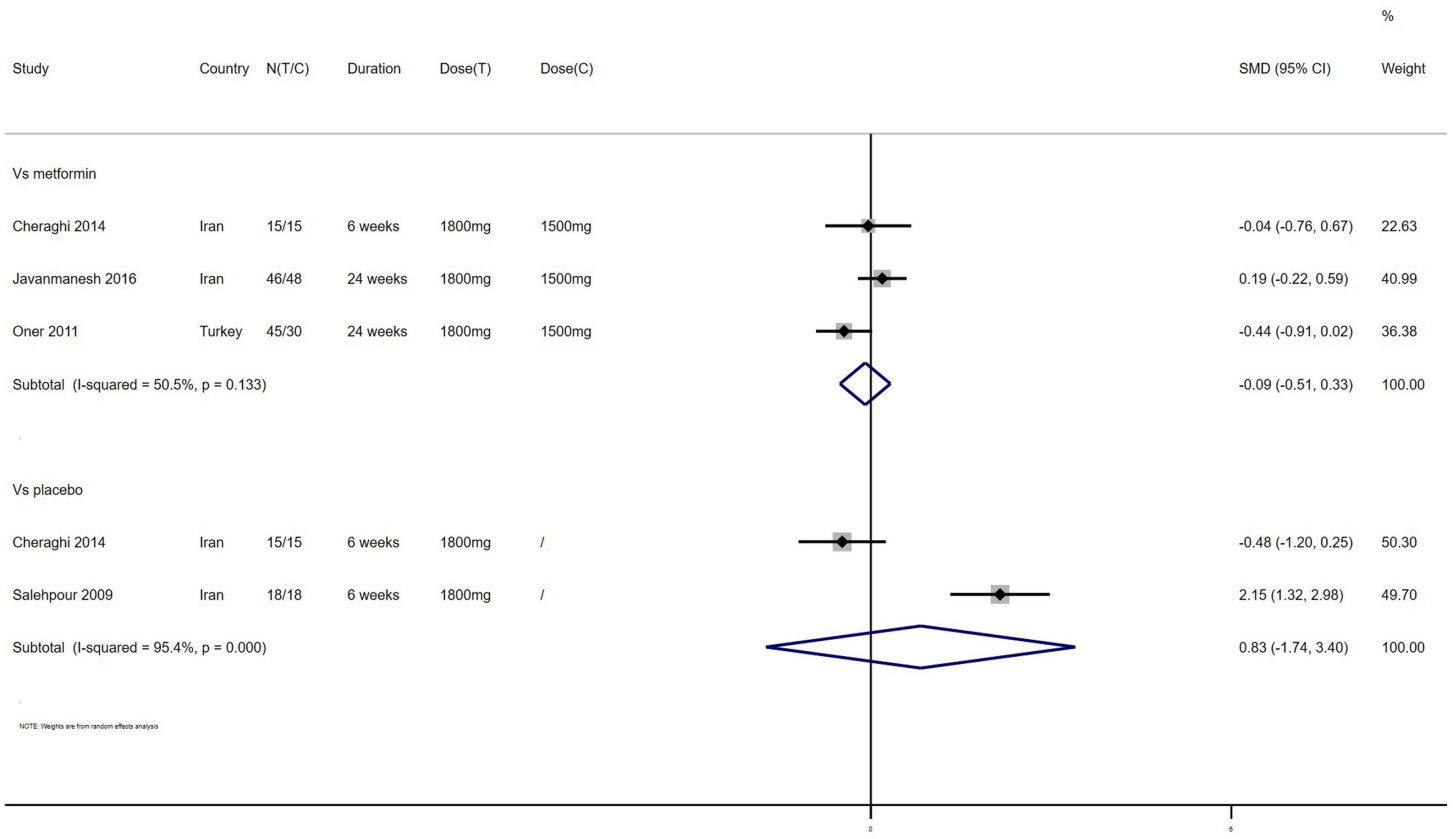
Forest plots of comparing NAC with metformin or placebo on low density lipoprotein (LDL).

LDL – 4 studies reported the effect of NAC on LDL (Figure 9). The results suggested that NAC reduced LDL levels in women with PCOS compared with metformin (SMD: −0.09, 95% CI: −0.51 to 0.33, *I*^2^ = 50.5%, P_H_ = 0.133, *p* = 0.662). However, NAC increased LDL levels when compared with placebo (SMD: 0.83, 95% CI: −1.74 to 3.40, *I*^2^ = 95.4%, P_H_ = 0, *p* = 0.528).

**Figure 10:**
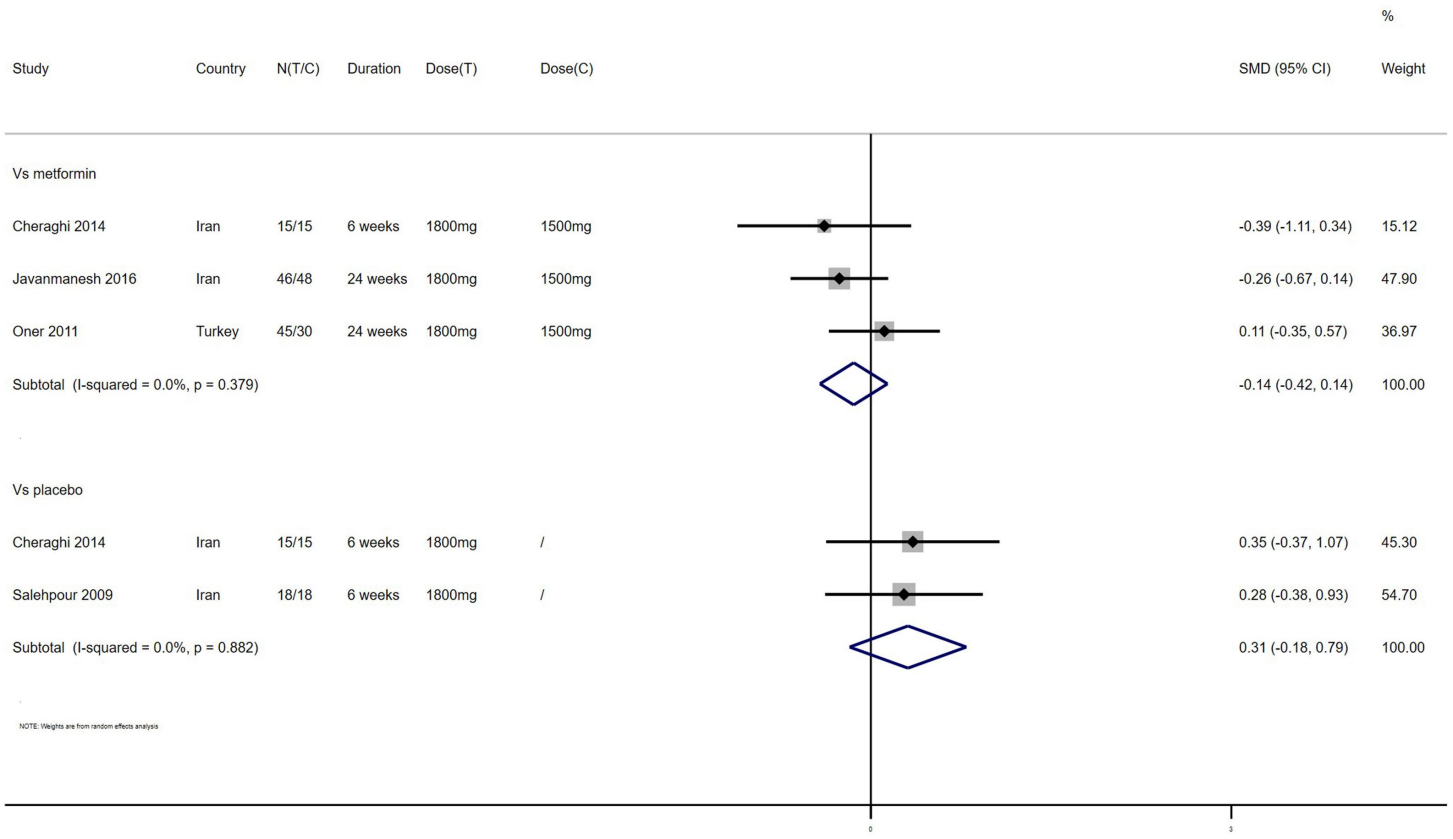
Forest plots of comparing NAC with metformin or placebo on high density lipoprotein (HDL).

HDL – 4 studies reported the effect of NAC on HDL (Figure 10). The results suggested that NAC increased HDL levels in women with PCOS compared with placebo (SMD: 0.31, 95% CI: −0.18 to 0.79, *I*^2^ = 0, P_H_ = 0.882, *p* = 0.212). However, NAC reduced HDL levels when compared with metformin (SMD: −0.14, 95% CI: −0.42 to 0.14, *I*^2^ = 0, P_H_ = 0.379, *p* = 0.321).

### Results of subgroup analyses

#### Results of subgroup analyses

Subgroup analyses of BMI, FBG, and FI were conducted based on the degree of obesity, study region, and follow-up time. Compared with obese PCOS women, NAC may be more beneficial to the improvement of BMI and FI in overweight PCOS women, but the difference is slight and not statistically significant. The region-based results showed that NAC could significantly reduce BMI, FBG and FI compared with metformin in South Asian women with PCOS (SMD: −0.39, 95% CI, −0.60 to −0.18, *p* < 0.001; SMD: −0.34, 95% CI, −0.53 to −0.15, *p* < 0.001; SMD: −0.59, 95% CI, −0.98 to −0.20, *p* = 0.003, respectively). We included studies with follow-up durations ranging from 6 to 24 weeks. The results suggested that the reduction of BMI, FBG and FI levels may be positively correlated with the duration of NAC intervention. Compared with metformin, NAC significantly reduced the levels of BMI, FBG and FI after 24 weeks of follow-up (SMD: −0.36, 95% CI, −0.62 to 0.80, *p* = 0.008; SMD: −0.51, 95% CI: −0.73 to −0.30, *p* < 0.001; SMD: −0.62, 95% CI: −1.11 to 10.13, *p* = 0.014). Detailed subgroup analyses are available in Table 2.

**Table 2:** Results of subgroup analyses of BMI, FBG, and FI.

Subgroup	No. of studies	No. of participants		SMD (95% CI)	*I* ^2^	*P*_Q_	*p*
		NAC	MET				
**Subgroup of body mass index**							
*BMI*							
24 < BMI < 28	5	182	182	−0.20 (−0.43, 0.02)	24.5%	0.250	0.078
28 < BMI	4	195	182	−0.17 (−0.73, 0.39)	87.5%	<0.001	0.548
*Region of study*							
South Asia	5	217	217	−0.39 (−0.60, −0.18)	14.5%	0.322	<0.001
Middle East	5	191	178	0.03 (−0.31, 0.37)	69.6%	0.006	0.874
*Duration of intervention*							
6 Weeks	2	46	46	0.15 (−0.26, 0.56)	0.0%	0.948	0.470
8 Weeks	1	54	54	0.10 (−0.28, 0.48)	/	/	/
12 Weeks	4	176	176	−0.15 (−0.69, 0.39)	84.2%	<0.001	0.591
24 Weeks	4	186	173	−0.36 (−0.62, 0.80)	37.0%	0.190	0.008
**Subgroup of fasting blood glucose**							
*BMI*							
24 < BMI < 28	5	182	182	−0.21 (−0.53, 0.12)	63.9%	0.017	0.208
28 < BMI	4	195	182	−0.23 (−0.53, 0.08)	59.8%	0.059	0.150
*Region of study*							
South Asia	5	217	217	−0.34 (−0.53, −0.15)	0.0%	0.406	<0.001
Middle East	5	191	178	−0.11 (−0.44, 0.22)	67.3%	0.009	0.513
*Duration of intervention*							
6 Weeks	2	46	46	0.39 (−0.03, 0.80)	0.0%	0.494	0.066
8 Weeks	1	54	54	0.08 (−0.29, 0.46)	/	/	/
12 Weeks	4	176	176	−0.23 (−0.43, −0.02)	0.0%	0.649	0.032
24 Weeks	4	186	173	−0.51 (−0.73, −0.30)	0.0%	0.748	<0.001
**Subgroup of fasting insulin**							
*BMI*							
24 < BMI < 28	5	182	182	−0.30 (−0.86, 0.26)	87.7%	<0.001	0.295
28 < BMI	4	195	182	−0.18 (−0.43, 0.07)	39.6%	0.174	0.166
*Region of study*							
South Asia	5	217	217	−0.59 (−0.98, −0.20)	75.6%	0.003	0.003
Middle East	5	191	178	0.06 (−0.23, 0.34)	56.8%	0.041	0.693
*Duration of intervention*							
6 Weeks	2	46	46	0.42 (−0.35, 1.19)	67.5%	0.080	0.285
8 Weeks	1	54	54	−0.00 (−0.38, 0.37)	/	/	/
12 Weeks	4	176	176	−0.18 (−0.40, 0.05)	14.7%	0.319	0.125
24 Weeks	4	186	173	−0.62 (−1.11, 10.13)	80.8%	0.001	0.014

## Discussion

### Main findings

This systematic review and meta-analysis included 11 RCTs involving 869 women with PCOS and found that NAC, like metformin, could improve metabolic disorders in women with PCOS. NAC significantly reduced FBG levels compared with metformin and TC levels compared with placebo. The results of subgroup analyses showed that long-term NAC intervention may be more beneficial to the improvement of BMI, FBG and FI in PCOS women. NAC significantly improved BMI, FBG and FI in women with PCOS in India.

Metformin, as an insulin sensitizer, is commonly used in PCOS with insulin resistance (IR). However, long-term metformin use may result in hypoglycemia, gastrointestinal dysfunction, vitamin B12 deficiency, and hyperhomocysteinemia (HCY) (33). HCY may be related to an increased risk of metabolic syndrome and CVD events (34). In our included study, 2 patients withdrew from treatment because they could not tolerate the gastrointestinal side effects of metformin (23). NAC also improved insulin sensitivity and was well tolerated with few side effects (18). A 12-week study reported more side effects with metformin than with NAC in women with PCOS, mainly characterized by a higher incidence of headache, nausea, and diarrhea (25). Another study also showed that NAC showed better tolerability compared to metformin (15% vs. 10%) (29). Therefore, NAC may be an alternative to metformin for the treatment of metabolic disorders in women with PCOS. However, the side effects of NAC are only known from short-term follow-up studies (6–24 weeks), so further evaluation of the long-term safety of NAC in women with PCOS is warranted. Studies have shown that NAC is well tolerated and remains non-toxic at concentrations where cysteine is considered potentially toxic (35). NAC releases cysteine very slowly, thus avoiding the side effects of acute exposure of human tissues to super physiological concentrations of cysteine (36, 37). In other words, NAC can feed cells with the slow release of cysteine over a long period of time.

Among metabolic disorders in women with PCOS, dyslipidemia is the most persistent and prevalent (38). Healthy lifestyle intervention does not significantly improve the blood lipid level in women with PCOS, and the effect of insulin sensitizers on dyslipidemia in women with PCOS is similar to that of lifestyle intervention (38). Metformin alone still has an unsatisfactory impact in treating dyslipidemia in obese PCOS women. In obese PCOS women, HDL decreased in the third decade of the whole lifespan, and TG began to rise in the second decade. Therefore, early prevention of dyslipidemia may have more clinical significance (38). Our evidence based on a limited number of publications suggests that NAC may be more beneficial than metformin in improving dyslipidemia in women with PCOS.

Subgroup analyses based on follow-up time showed that longer duration of NAC intervention was associated with more effective changes in BMI, FBG and FI, and longer duration of NAC treatment was associated with better outcomes. This is consistent with the conclusions of the two included studies (28, 29). PCOS is a common condition with important effects throughout the life cycle, including reproductive, metabolic, and psychological effects, so long-term interventions are important in PCOS management (39). The long-term use of NAC may meet the management goals of PCOS. South Asian PCOS women’s use of NAC was more effective in improving BMI, FBG, and FI compared with Middle Eastern PCOS women. This may be due to differences in ethnicity, environment and PCOS phenotype among the participants (40). However, the number of countries included in the subgroup analysis is limited and may not reflect the overall level in the region, so this conclusion should be interpreted with caution. In addition, the countries included in the analysis were all Asian and African countries, which may limit the generalizability of our conclusions. Subgroup analysis based on BMI showed that NAC was more effective in overweight women (24 < BMI < 28) with PCOS compared with obese women (BMI > 28). This may be related to more mild metabolic disorders in overweight PCOS women. Studies have reported that women with PCOS who lose 5% of their body weight have improved menstrual irregularities, ovulation disorders, and metabolic disorders (39).

### Possible mechanisms of NAC treatment for PCOS

Metabolic syndrome may be caused by abdominal obesity, which is associated with hyperinsulinemia and IR in PCOS women (41). Although IR is not included in the diagnostic criteria of PCOS, it is very common in PCOS women, and about 95% of obese PCOS women have IR (2, 42). With regard to the mechanism of IR in PCOS women, it’s possible that as oxidative stress rises, a number of protein kinases become active, which then cause the deterioration of insulin receptor substrates and finally the formation of IR. (43). According to published studies, oxidative stress may contribute to the pathophysiology of PCOS, and elevated levels of oxidative stress are linked to hyperinsulinemia and dyslipidemia. (44). Therefore, antioxidants may be beneficial in improving metabolic disorders in women with PCOS, which was demonstrated in another study (45). NAC can improve metabolic parameters in women with PCOS, which may be related to its antioxidant effect. Second, NAC supplementation can improve the levels of interleukin-6, malondialdehyde, and homocysteine, which may have a positive effect on metabolism (46–49). Third, a framework for a new mechanism of action is emerging in which NAC acts as a prodrug of cysteine, leading to modest elevations of hydrogen sulfide and sulfathionate within cells. In addition, the slow release of cysteine in NAC allows the production of sulfone sulfur. The tissue protection provided by these substances can be independent of glutathione supplementation and may therefore improve metabolic disorders in patients with PCOS (35).

### Limitations

Our study has the following limitations. First, only a small number of publications reported the impact of NAC on the lipid indicators of PCOS, so subgroup analyses could not be performed. In addition, only 2 studies were related to placebo studies, which to some extent may affect the reliability of the results. Second, the duration of the NAC intervention was short (6–24 weeks), although the results suggest that the effect of NAC may be positively correlated with the duration of the intervention. A longer follow-up duration is still necessary to assess the long-term efficacy and safety of NAC. Third, the number of participants was small and most were conducted in Asian countries, so the conclusions may not be generalizable to all types of women with PCOS. In addition, PCOS has different phenotypes according to the Rotterdam diagnostic criteria, and we could not provide additional evidence regarding the effect of NAC on different phenotypes. Fourth, most studies did not report randomization and allocation concealment, which may have affected the overall quality of the included literature. However, it has been suggested that the lack of adequate information on randomization and allocation concealment may be a reporting problem in the literature rather than a real flaw in the study (50).

### Implications for practice

PCOS has a higher risk of cardiovascular risk factors, and the American College of Cardiology and American Heart Association guidelines consider PCOS to be a risk enhancing factor for CVD (51). Therefore, long-term health intervention has important clinical significance for the prevention of CVD in the management of PCOS. Recent international guidelines for the management of PCOS recommend regular testing of metabolic indicators such as blood sugar and blood lipids, and indicate that nutritional supplements such as inositol are experimental treatments for PCOS which may benefit PCOS (52). NAC, as a nutritional supplement, has antioxidant function and can improve the oxidative stress state, which may be a potential drug for the therapy of PCOS. Second, there is inadequate evidence regarding the impact of NAC on the metabolism of different phenotypes of PCOS. Therefore, well-designed RCTs with large sample sizes can be performed to evaluate the effect of NAC on different phenotypes of PCOS.

## Conclusion

This study on the treatment of PCOS provides additional evidence that NAC may be a potential therapeutic intervention for PCOS. Like metformin, NAC can improve metabolic disorders in women with PCOS and is well tolerated, which may be more conducive to the long-term management of PCOS.

## Data availability statement

The original contributions presented in the study are included in the article/Supplementary material, further inquiries can be directed to the corresponding author.

## Author contributions

JL and HS designed the study protocol and writing the manuscript. HS, LW, and XJ literature search, data extraction, and data analysis. XJ drew figures and tables. JH reviewed the study process, and had primary responsibility for the final manuscript. All authors contributed to the article and approved the submitted version.

## Funding

This study was funded by the Ouyang Huiqing Studio of Famous Traditional Chinese Physicians from the Administration of Traditional Chinese Medicine (no. 2018119).

## Conflict of interest

The authors declare that the research was conducted in the absence of any commercial or financial relationships that could be construed as a potential conflict of interest.

## Publisher’s note

All claims expressed in this article are solely those of the authors and do not necessarily represent those of their affiliated organizations, or those of the publisher, the editors and the reviewers. Any product that may be evaluated in this article, or claim that may be made by its manufacturer, is not guaranteed or endorsed by the publisher.
