# Supplementary material for: The effects of N-acetylcysteine supplement on metabolic parameters in women with polycystic ovary syndrome: a systematic review and meta-analysis
Source: Front Nutr. 2023 Sep 29;10:1209614. doi: 10.3389/fnut.2023.1209614 (PMC10573309; doi:10.3389/fnut.2023.1209614)
Supplement: Supplementary file 1 [file Table_1.DOCX]

**Detailed search strategy**

PUBMED (Searched on: April 6, 2023)

| Search number | Query | Results | Time |
| --- | --- | --- | --- |
| #1 | ((((Acetylcysteine[MeSH Terms]) OR (Acetylcysteine[Title/Abstract])) OR (Nacetylcysteine[Title/Abstract])) OR (N-Acetylcysteine[Title/Abstract])) OR (NAC[Title/Abstract]) Sort by: Most Recent | 38,446 | 18:30:03 |
| #2 | (((((((polycystic ovarian syndrome[MeSH Terms]) OR (polycystic ovarian syndrome[Title/Abstract])) OR (Polycystic Ovary Syndrome[Title/Abstract])) OR (Ovary Syndrome[Title/Abstract])) OR (Polycystic Syndrome[Title/Abstract])) OR (Polycystic Ovary[Title/Abstract])) OR (Polycystic ovary disease[Title/Abstract])) OR (PCOS[Title/Abstract]) Sort by: Most Recent | 23,759 | 18:32:22 |
| #3 | ((((Randomized controlled trial[Publication Type]) OR (Randomized controlled trial[Title/Abstract])) OR (Randomized[Title/Abstract])) OR (Placebo[Title/Abstract])) OR (Random[Title/Abstract]) Sort by: Most Recent | 1,304,337 | 18:33:01 |
| #4 | #1 AND #2 AND #3 | 24 | 18:34:55 |

EMBASE (Searched on: April 6, 2023)

| Search number | Query | Results | Time |
| --- | --- | --- | --- |
| #1 | 'acetylcysteine'/exp OR acetylcysteine:ti,ab,kw OR nacetylcysteine:ti,ab,kw OR 'n acetylcysteine':ti,ab,kw OR nac:ti,ab,kw | 65,798 | 19:12:11 |
| #2 | 'ovary polycystic disease'/exp OR 'polycystic ovarian syndrome':ti,ab,kw OR 'polycystic ovary syndrome':ti,ab,kw OR 'ovary syndrome':ti,ab,kw OR 'polycystic syndrome':ti,ab,kw OR 'polycystic ovary':ti,ab,kw OR 'polycystic ovary disease':ti,ab,kw OR pcos:ti,ab,kw | 40,706 | 19:17:23 |
| #3 | 'randomized controlled trial'/exp OR 'randomized controlled trial':ab,ti OR 'rct':ab,ti OR 'random':ab,ti OR 'placebo':ab,ti | 1,735,490 | 19:18:13 |
| #4 | #1 AND #2 AND #3 | 57 | 19:22:09 |

WOS (Searched on: 30 December 2022)

| Search number | Query | Results | Time |
| --- | --- | --- | --- |
| #1 | (((TS=(Acetylcysteine)) OR TS=(Nacetylcysteine)) OR TS=(N-Acetylcysteine)) OR TS=(NAC) | 38,522 | 20:32:07 |
| #2 | ((((((TS=(polycystic ovarian syndrome)) OR TS=(Polycystic Ovary Syndrome)) OR TS=(Ovary Syndrome)) OR TS=(Polycystic Syndrome)) OR TS=(Polycystic Ovary)) OR TS=(Polycystic ovary disease)) OR TS=(PCOS) | 28.924 | 20:38:42 |
| #3 | ((((TS=(randomized controlled trial)) OR TS=(RCT)) OR TS=(random)) OR TS=(randomized)) OR TS=(placebo) | 1,606,810 | 20:50:37 |
| #4 | #1 AND #2 AND #3 | 22 | 20:58:22 |

Cochrane (Searched on: 30 December 2022)

| Search number | Query | Results | Time |
| --- | --- | --- | --- |
| #1 | ("acetylcysteine"):ti,ab,kw OR (Nacetylcysteine):ti,ab,kw OR (N-Acetylcysteine):ti,ab,kw OR (NAC):ti,ab,kw | 3,654 | 21:33:22 |
| #2 | (polycystic ovarian syndrome):ti,ab,kw OR (Polycystic Ovary Syndrome):ti,ab,kw OR (Ovary Syndrome):ti,ab,kw OR (Polycystic Syndrome):ti,ab,kw OR (Polycystic Ovary):ti,ab,kw OR (Polycystic ovary disease):ti,ab,kw OR (PCOS):ti,ab,kw | 5,806 | 21:38:42 |
| #4 | #1 AND #2 | 44 | 21:48:24 |
