# Supplementary material for: The effects of N-acetylcysteine supplement on metabolic parameters in women with polycystic ovary syndrome: a systematic review and meta-analysis
Source: Front Nutr. 2023 Sep 29;10:1209614. doi: 10.3389/fnut.2023.1209614 (PMC10573309; doi:10.3389/fnut.2023.1209614)
Supplement: Supplementary file 2 [file Table_2.DOCX]

Risk of bias graph for randomized controlled trials.

| **Study** | **Random sequence generation** | **Allocation concealment** | **Blinding of participants and personnel** | **Blinding of outcome date** | **Incomplete outcome assessment** | **Selective repoting** | **Other bias** |
| --- | --- | --- | --- | --- | --- | --- | --- |
| Arya 2022 | low | unclear | low | high | low | low | low |
| Chandil  2018 | unclear | unclear | low | high | low | low | low |
| Cheraghi  2014 | unclear | low | low | unclear | low | low | low |
| Elnashar  2007 | low | unclear | low | unclear | low | low | low |
| Gayatri  2010 | low | unclear | low | low | low | low | low |
| Gupta  2017 | unclear | unclear | low | unclear | low | low | low |
| Javanmanesh  2015 | unclear | low | low | unclear | low | low | low |
| Kumar  2018 | low | low | low | high | low | low | low |
| Nemati  2017 | low | unclear | low | unclear | low | low | low |
| Oner  2011 | unclear | low | low | low | low | low | low |
| Salehpour  2009 | unclear | unclear | low | low | low | low | low |

low: low risk of bias; high: high risk of bias; unclear: unclear risk of bias.
